# Feasibility and Safety Study of the Use of a New Robot (Maestro™) for Laparoscopic Surgery

**DOI:** 10.1007/s11695-024-07409-9

**Published:** 2024-08-01

**Authors:** Guy-Bernard Cadière, Jacques Himpens, Mathilde Poras, Nicolas Boyer, Benjamin Cadière

**Affiliations:** 1 Digestive Surgery, Hôpital Universitaire St Pierre, ULB, Brussels, Belgium; 2 Digestive Surgery Institut Tzanck, Saint-Laurent-du-Var, France; 3 CHU St Pierre, Brussels, Belgium

**Keywords:** Robotic-assisted surgery, Laparoscopic surgery, First in human, Cholecystectomy, Colectomy, Obesity surgery

## Abstract

**Background:**

In laparoscopic surgery, telerobotic systems such as Da Vinci™ were developed, among other things, to give back exposure and vision control to the operating surgeon. However, new limitations such as the separation of the operating surgeon from the operating table, cost, and size were unveiled. A new device, Maestro™, appears promising in addressing these limitations. The current work evaluates the feasibility, safety, and surgeon satisfaction with the assistance provided by the Maestro System.

**Methods:**

Non-consecutive patients who were candidates for laparoscopic digestive surgery were enrolled in a descriptive prospective, monocentric study. Case selection was solely based on the availability of the device, but not on the patient’s characteristics. Surgery was performed by a leading surgeon with the help of one less experienced surgeon. Feasibility was defined by the maintenance of the initial surgical plan. Safety was assessed by the absence of serious adverse events related to the device and surgeon satisfaction was evaluated by a questionnaire following the intervention.

**Results:**

All 50 procedures were completed without conversion in laparotomy and without adjustment of the surgical team. Four complications were recorded during the study; however, none related to the use of the Maestro System. In 92% of the cases, the surgeon was satisfied with the assistance provided by the Maestro System.

**Conclusions:**

In standard elective digestive procedures by laparoscopy, the use of the Maestro System is feasible and safe. It is beneficial to the surgeon and operative room organization by limiting the size of the surgical team.

**Graphical Abstract:**

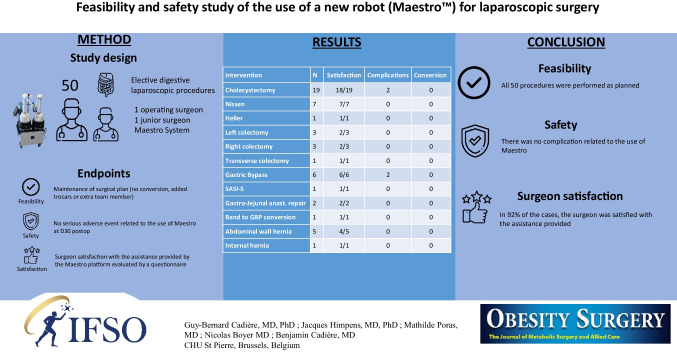

## Introduction

With over 13 million procedures [1] performed over the globe each year, laparoscopic surgery constitutes the standard of care. However, a major challenge in laparoscopic surgery consists of the inherent delegation of vision and exposure control to third-party individuals such as residents, interns, or operating assistants. If these individuals are not skilled enough, the critical view of safety is not obtained.

Several telerobotic systems have been developed to give back exposure and vision control to the operating surgeon These systems take motions from a non-sterile surgeon and translate those into mirrored motions in the sterile field at the bedside. Pioneering systems include the Da Vinci (Intuitive Surgical, Sunnyvale, CA) [2]; more recently, other innovative robotic systems such as the HUGO™ robotic-assisted surgery (RAS) system (Medtronic) and the Versius™ (CMR) have been introduced [3–5]. These telerobotic systems are mainly engineered to enhance the surgeon’s control and expand the degrees of freedom of ancillary tools during surgery [6].

The adoption of telerobotic systems is impacted by several factors. Compared to conventional laparoscopy, the operating time, including setup duration [7], is prolonged [8], especially when a dedicated robot staff with specific training is unavailable, and the operating room size is not adapted. The high cost of acquiring, using, and maintaining telerobotics has prevented its large-scale adoption [9, 10]. The lack of tactile “force” feedback can be a detrimental limitation of telerobotics. Lastly, the separation of the operating surgeon from the operating table and sterile environment reduces operative awareness and restricts communication to verbal commands between surgeons, assistants, nurses, and anesthesiologists.

To eliminate the complexity associated with telerobotics, another field of robotic-assisted surgery was developed: collaborative robotics. In telerobotics, the system performs the procedure through the control of the surgeon, whereas in collaborative robotics, the system assists the surgeon in performing the procedure through collaborative control of the instruments at the bedside.

Pioneering collaborative robotics, a new system, Maestro (Moon Surgical, Paris, France), was developed to provide the control enjoyed by telerobotics without the loss of the sense of touch, the surgeon’s absence at the bedside, and impact on operative time experienced with telerobotics.

After extensive training on 30 cadaver models, ten patients eventually underwent laparoscopic cholecystectomy using the Maestro System [11]. The thorough and unbiased assessment of all the details observed in this small cohort constituted the cornerstone for further in-vivo development of this collaborative robotic system [11].

Because the size of the operative field is quite limited in laparoscopic cholecystectomy, it was imperative to establish the benefit of the system in different surgical indications involving wider operative fields.

Consequently, the objective of the current work was to evaluate the feasibility, safety, and satisfaction of the surgeon with the assistance provided by the Maestro System in standard elective minimally invasive digestive laparoscopic procedures.

## Method

### Study Design

Following a thorough evaluation of feasibility studies on the cadavers of thirty consenting individuals, the “First-in-Human” phase was initiated. This phase encompassed a prospective, monocentric study conducted at Hôpital St-Pierre, Brussels. The study was duly registered under the identifiers CIV-22–01-038769 (Eudamed) and NCT05243433 (clinicaltrials.gov). The study was registered by the FAMHP (Federal Agency for Medicines and Health Products) with the approval of the local Ethics Committee.

All surgeries were performed by a team consisting of two operators: a senior surgeon (GBC) and a junior surgeon (LP or MP). The senior is a general surgeon with more than 10,000 lap surgeries including colorectal, upper GI, parietal, and obesity surgery. He is one of the pioneers of robotic surgery [2] [12]. The junior surgeon (LP or MP) did not have an extensive experience in laparoscopic procedures before their proctoring with the Maestro System. In most cases, the senior surgeon was the operating individual, and in some easier cases, the operator was the junior, assisted by the senior.

The patients were non-consecutive candidates for standard laparoscopic surgery. They had signed a fully informed consent form. The inclusion criteria for the study were as follows: patients aged of more than 18 years, who were scheduled for standard elective laparoscopic digestive procedures such as cholecystectomy, hernia repair, appendectomy, bariatric surgery (either sleeve gastrectomy or Roux-en-Y gastric bypass), or colectomy. The exclusion criteria were as follows: individuals with active systemic or cutaneous infection or inflammation, anemia defined as Hb < 10 g/dl or hematocrit less than 30%), women if pregnant or breastfeeding, and patients with obesity with BMI exceeding 45 kg/m^2^.

There was no selection based on patient characteristics, as long as they entered the inclusion criteria. The study cases were limited by Moon Surgical’s team availability, as they had to travel from abroad.

Primary endpoints concerned feasibility and safety of the procedures using the Maestro System. Feasibility was defined by the maintenance of the initial procedural plan, which means there is no need for additional trocars, additional team members, and conversion to conventional laparoscopy or to open surgery caused by failure or malfunction of any type of the Maestro System. Safety was assessed by the absence of device-related adverse events during the procedure and within the ensuing 30 days.

The secondary endpoint was the satisfaction of the surgeon with the assistance provided by the Maestro System. Satisfaction was assessed by a questionnaire for the surgeon following the intervention. Surgeon’s satisfaction refers to the operating surgeon, i.e., the senior surgeon on most occasions, or the junior when he/she was the operator.

Assessment by the questionnaire allowed to grade surgeon satisfaction with the assistance provided by the Maestro System as follows: very dissatisfied, TI; dissatisfied, I; neither dissatisfied nor satisfied, NINS; satisfied, S; very satisfied, TS. The % of cases in which surgeon’s satisfaction was graded (S) to very satisfied (TS) was defined as the satisfaction index.

Patient demographic data was collected, including age, sex, BMI, and history of abdominal surgery.

Peroperative data was collected as well: duration of the procedure, possible adverse event(s) occurring during the procedure, and atypical anatomy of the patient (identified by imaging and peroperative observation). In these latter instances, a detailed description of the anatomy was recorded, which allowed to provide a comprehensive insight into any variables with possible implications on the procedure itself or the outcomes using the Maestro System.

Additional peroperative operative data collected included the following: difficulty of the surgical procedure as perceived by the surgeon: very easy, TF; easy, F; moderate, M; difficult, D; impossible, I.

Postoperative data collected per patient included the following: pain evaluation at 24 h (visual analog scale, VAS, 0–10), number of rehospitalizations within 30 days, number of reoperations within 30 days, and presence of adverse events within 30 days postoperatively as assessed by telephone contact. Adverse events were graded on the Clavien-Dindo scale [13].

### Statistical Analysis

Data collected during the study was analyzed to determine statistical values.

For patient demographics, the average value was calculated (Table 1). For peroperative data, such as operative length, and postoperative stay, the median value was calculated. For each median or average value of a statistical series, the standard deviation was calculated as well.

**Table 1:** Demographic data

Features	Value
Total	50
Average age, years	54.05 ± 13.71 (21–92)
Male ratio, *n* (%)	15 (30%)
Average BMI, kg/m^2^	27.88 ± 5.26 (17.19–54.56)

### Maestro System

The Maestro™ System (Moon Surgical, Paris, France) is a robotic system with two articulated co-manipulated arms which hold the optical system and another laparoscopic instrument intended for exposure during laparoscopic surgery.

**Fig. 1:**
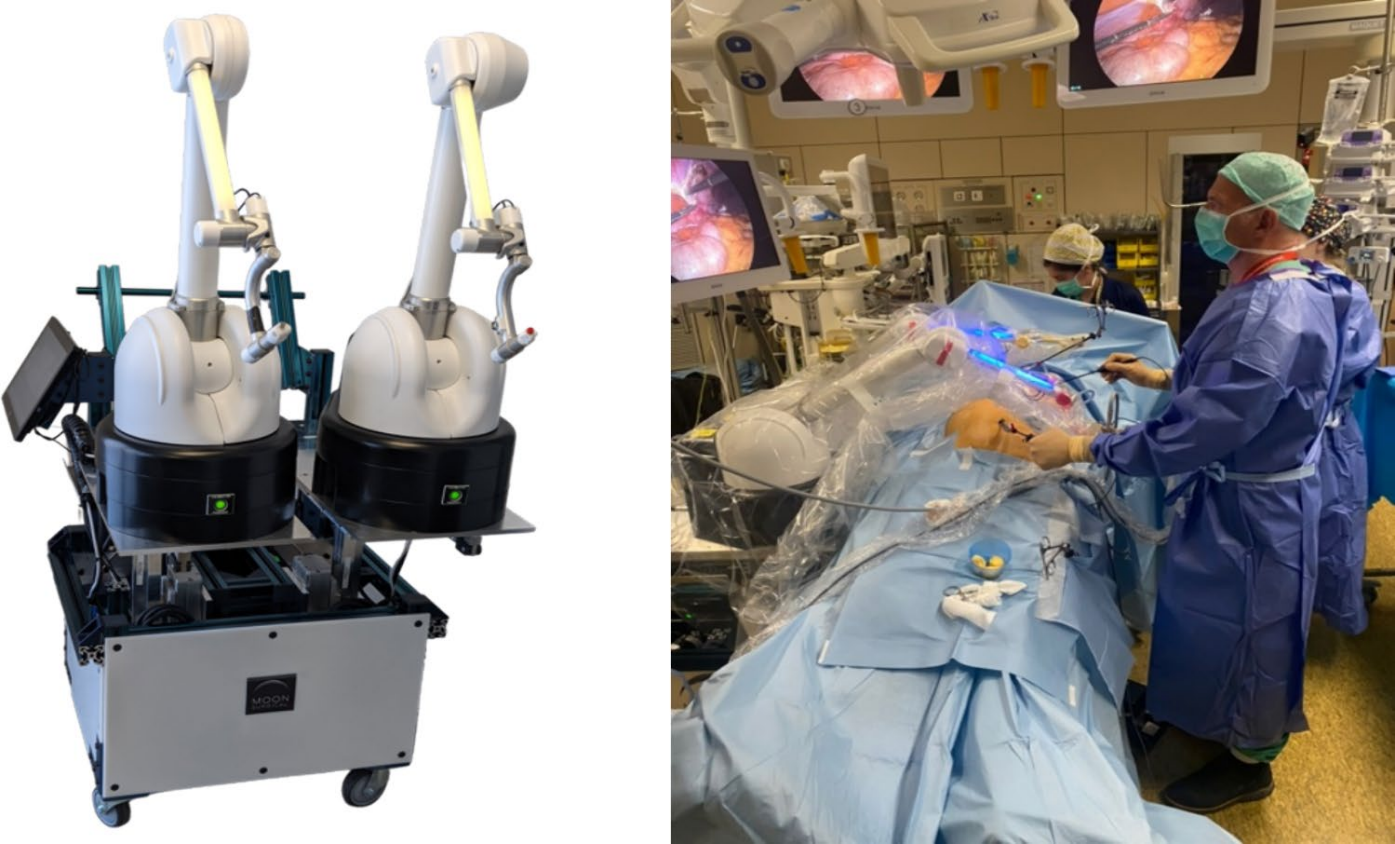
The Maestro System

The two arms of the Maestro System (Fig. 1) have been designed to minimize mechanical friction and to constantly compensate for the weight of the optical system and the force exerted on the retraction clamp.

The combination of these two factors allows us to manipulate and reposition instruments with total transparency.

### Surgical Technique

**Fig. 2:**
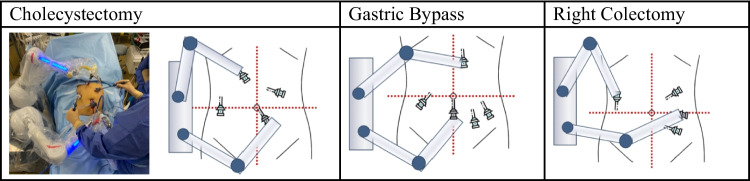
Maestro system configurations

After the patient had been installed, the pneumoperitoneum was created and the trocars and instruments were positioned, as in conventional laparoscopy (Fig. 2). The system was rolled to the operating table, with the arms positioned depending on the type of procedure.

The number and positioning of trocars were the same as for the conventional laparoscopic approach. The surgical team invariably consisted of two operators (GBC and one junior surgeon).

## Results

The 50 operations were carried out non-consecutively, depending on Moon Surgical staff availability, between April 13, 2022, and April 3, 2023.

One patient was included in the study despite being outside the inclusion criteria (high BMI).

### Intraoperative Data

All 50 operations were successfully performed as defined in the feasibility criteria including the consistency of the surgical team.

In all the cases in the study, the surgeon's satisfaction index was 92%. The operation was considered difficult in 8% of cases and with atypical anatomy in 34% of cases. Table 2 displays the numbers of each intervention type, the median intervention time, the number of atypical anatomies, the number of difficult procedures, the satisfaction index, and the number of peroperative complications.

**Table 2:** Peroperative data

Types of intervention	Number	Median intervention time (min)	Atypical anatomy	Difficult procedure	Satisfaction index (TS-S)	Peroperative complications
Cholecystectomy	19	42 (± 32.09)	6	3/19 (15.8%)	18/19 (94.7%)	1
Nissen	7	58(± 24.43)	3	1/7 (14.28%)	7/7 (100%)	0
Heller	1	96	0	0/7 (0%)	1/1 (100%)	0
Left colectomy	3	183(± 35.30)	1	0/3 (0%)	2/3 (66.6%)	0
Right colectomy	3	138(± 8.62)	1	0/3 (0%)	2/3 (66%)	0
Transverse colectomy	1	113	0	0/1 (0%)	1/1 (100%)	0
Gastric bypass	6	93(± 35.11)	2	0/6 (0%)	6/6 (100%)	0
SASI-S	1	82	0	0/1 (0%)	1/1 (100%)	0
Gastro-jejunal anastomosis repair	2	93(± 43.84)	2	1/2 (50%)	2/2 (100%)	0
Band to GBP conversion	1	163	1	0/1 (0%)	1/1 (100%)	0
Abdominal wall hernia	5	50(± 26.36)	0	0/5 (0%)	4/5 (80%)	0
Internal hernia	1	36	1	0/1 (0%)	1/1 (100%)	0
Total	50	71	17/50 (34%)	5/50 (10%)	46/50 (92%)	1/50 (2%)

#### Cholecystectomy (19 Cases)

Ten of these 19 cholecystectomies were presented in a previous article [11]. The median duration was 42 min (32.09 standard deviation). In 94.7% (18/19) of the surgeries, the surgeon was satisfied to very satisfied with the robot’s assistance. In one case, the surgeon was not satisfied; i.e., in the first case out of the 50, the magnet’s fixing force was not sufficient causing the optical system to detach on multiple occasions. Atypical anatomies were noted in six cases: in five cases, there were adhesions or fibrosis at Calot’s triangle, and in one case, a periaortic hematoma occurred by puncture of the Veress needle during initiation of pneumoperitoneum. This latter patient underwent a repeat laparoscopic operation the following day. This intraoperative complication (Clavien-Dindo Grade IIIb) was not related to the Maestro System. The procedure was considered difficult by the surgeon in three cases: in one, the gallbladder measuring 12.7 × 6 cm was associated with Mirrizi syndrome. In the second case, the gallbladder was fibrotic and displayed adherences with the Calot’s triangle. In the third case, the gallbladder was severely inflamed and presented adhesions. In the three cases mentioned, the surgeon was very satisfied (TS) with the assistance provided by the Maestro System.

#### Nissen (7 Cases)

The median duration was 58 min (24.43 standard deviation), and the satisfaction index was 100%. Atypical anatomies occurred in three cases: two cases of undo Nissen-Nissen procedure and one case of fibrosis of the esogastric junction. One procedure was considered difficult.

#### Left Colectomy (3 Cases)

The colorectal anastomosis was termino-terminal with a circular stapler. The median duration was 183 min (35.30 standard deviation). The operative time of the three left colectomies was 210, 183, and 140 min respectively. The satisfaction index was 66.6% (2/3). In the first case of left colectomy, the surgeon was dissatisfied with the assistance provided by the Maestro System, even though the procedure was not considered difficult, and the anatomy was not considered atypical. The dissatisfaction was due to conflicts and interference of the articulated arms due to the non-optimal preoperative positioning of the Maestro System. There was one occurrence of atypical anatomy. No procedure was considered difficult by the surgeon.

#### Right Colectomy (3 Cases)

The ileo-colic anastomosis was lateral and intracorporeal. The median duration was 138 min (8.62 standard deviation). The surgeon’s satisfaction index was 66%. There was one occurrence of atypical anatomy consisting of panniculitis of the greater omentum. No procedure was considered difficult by the surgeon.

#### Gastric Bypass (6 Cases)

The median duration was 93 min (35.11 standard deviation). The satisfaction index was 100%. There were two occurrences of atypical anatomy due to adhesions. No procedure was considered difficult by the surgeon.

#### Gastro-Jejunal Anastomosis Repair (2 Cases)

The median duration was 93 min (43.84 standard deviation). The satisfaction index was 100%. There was one occurrence of atypical anatomy due to a redo-surgery. One procedure was considered difficult by the surgeon.

#### Abdominal Wall Hernia (5 Cases)

The median duration was 50 min (26.36 standard deviation). The satisfaction index was 80%. There was one occurrence of atypical anatomy due to a difficult retro-perineal dissection. No procedure was considered difficult by the surgeon.

#### Reintervention (5 Cases)

In the five cases of revision (two gastro-jejunal anastomosis repairs, one conversion of an adjustable ring to gastric bypass, and two undo-Nissen-Nissen), the satisfaction index was 100%.

#### Proctoring (8 Cases)

In eight cases (six cholecystectomies, one Nissen fundoplication, one abdominal wall hernia repair), the primary surgeon was a young surgeon (LP, MP) assisted by the principal investigator (GBC). In these cases, there were no conversions to conventional laparoscopy and no complications. The young surgeons’ satisfaction with the assistance provided was 100%.

### Postoperative data

Three patients presented complications within 30 days:

A gastric bypass patient presented to the emergency department on day 7 post-intervention with abdominal pain. A CT scan showed a hematoma on a staple line. The patient was hospitalized for 4 days of monitoring (Clavien-Dindo Grade IIB). A gastric bypass patient presented with upstream dilation of the jejuno-jejunal anastomosis on day 3 post-intervention, requiring laparoscopic revision of the anastomosis. On day 6 post-intervention, the patient developed biliary peritonitis, necessitating revision of the anastomosis by laparotomy. A 5-day stay in intensive care was necessary (Clavien-Dindo Grade IV). Discharge was authorized on day 22. A cholecystectomy patient developed a biliary fistula on day 7 post-intervention, requiring re-hospitalization for biliary prosthesis.

On day 30 post-intervention, all patients were asymptomatic.

Table 3 here presents postoperative data.

**Table 3:** Postoperative data

Procedure	Number	VAS 24 h	Median hospital stays (days)	Post-operative complication
Cholecystectomy	19	1	1	1 rehospitalization for biliary fistula on postoperative day 7. Placement of a biliary prosthesis (Clavien grade IIIB)
Nissen fundoplication	7	5	1	
Heller	1	3	2	
Left colectomy	3	3	6	
Right colectomy	3	4	3	
Transverse colectomy	1	2	5	
Gastric bypass	6	3.5	2.5	1 4-day rehospitalization for monitoring of staple line hematoma (Clavien grade IIB)
				1 JJ anastomotic stenosis. Revision of anastomosis on D3. Biliary peritonitis with occlusion upstream of JJ anastomosis due to a large wall hernia. Laparotomy on PO day 6, ICU monitoring, hospital stay 22 days (Clavien grade IV)
SASI-S	1	0	3	
Band to GBP conversion	1	6	4	
G-J anastomosis repair	2	0.5	2.5	
Wall hernia	5	1	1	
Internal hernia	1	0	1	
Totals	50	2	1.5	3/50 (6%)

## Discussion

All 50 operations were successfully performed as defined in the method, even when the operation was considered difficult or the anatomy atypical. Of note, all procedures were carried out by just two operators (GBC assisted by a junior surgeon).

Among the 50 patients, there were four complications, two of which were unrelated to the use of the Maestro System. The first happened before the system was brought to the operating table, and the second consisted of a staple line bleed. For the remaining two complications, the procedure was judged difficult in one case (biliary fistula occurring after a cholecystectomy performed in a hostile surgical field) but not difficult in the other (fistula at the jejuno-jejunostomy during a gastric bypass). In gastric bypass, complications at the J-J anastomosis, however rare (less than 1% of cases [14]), do occur, even in experienced hands (GBC, with an experience of more than 2000 laparoscopic gastric bypasses, had already faced this complication.) Despite the occurrence of the complication, the senior surgeon — who was the operator — was satisfied with robot assistance. The complication was judged to be independent from the use of the robot, but rather to be related to a technical flaw that was not due to a particular visual issue such as poor exposure.

Hence, the primary objective was reached.

The Satisfaction Index (TS/S) was 92%, although 34% of the cases displayed the presence of atypical anatomy, and the perceived procedural difficulty reached 10% of the cases. Consequently, the secondary endpoint was reached. This favorable outcome can likely be attributed to several factors. Firstly, the resistance-free manipulation of instruments facilitated by the articulated arms enables the surgeon to maintain the same operative workflow. Secondly, the stability of the optical system ensures precise visualization throughout the procedure. Thirdly, the design of the system enables the surgeon to retain haptic feedback and to perceive the exerted force. Lastly, the trocar positioning and the use of off-the-shelf instruments further mimic the usual operative workflow.

In cholecystectomies (apart from the first case), esogastric junction surgeries (Nissen, Heller, gastric bypass, SASI), revision cases (post-band conversion, gastro-jejunal anastomosis revision, Undo-Nissen-Nissen), and right colectomy, operating time was similar to conventional laparoscopy, and the satisfaction index was 100%. Obesity and adhesions did not interfere with surgeon satisfaction. In the case of left colectomy, which involves a wide operative field, correct trocar placement was more difficult to establish, due to the conflict between the articulated arms. Nevertheless, after just three cases, the operating time was significantly reduced, and the satisfaction index improved.

Of note, retraction by the fenestrated forceps held by the Maestro System did not result in perforation of intestines or solid organs such as the liver.

In the case of abdominal wall hernias, the Maestro System seems to make less of a contribution, since a single articulated arm is used to hold the optical system.

Except for the left-sided colectomy, the learning curve was rapid as can be assessed by the satisfaction index and the operative time.

The median duration of the hospitalization for all the procedures was similar to the usual length of stay in our hospital.

Additionally, the proctoring process benefited from significant enhancement. The proctor surgeon was able to focus on instructing without constantly holding the optics and the exposure instruments. The proctoring surgeon could therefore correct the operative surgeon’s technique, hand them the instruments, and anticipate their needs, allowing the student to concentrate exclusively on the screen.

Limitations of this study were as follows: The patients’ cohort in the different procedures was rather small. The study was not randomized and did not benefit from a control group. Another risk of bias was the necessity of on-site presence of the Moon Surgical team which, unlike in everyday practice, facilitated the positioning and overall set-up of the system. The satisfaction evaluation of the surgeon was subjective and highly dependent on his personal background.

## Conclusion

Laparoscopic surgery suffers from a number of well-known shortcomings, including delegation of vision and exposure control. The Maestro System is a robotic platform aiming at addressing these specific shortcomings. The Maestro System was used successfully and safely by a restricted team of two surgeons in 50 unselected candidates for standard laparoscopic digestive surgeries. The surgeon’s satisfaction index with the assistance provided by the Maestro robot was 92%.
